# Moving from Empirical to Rational Vaccine Design in the ‘Omics’ Era

**DOI:** 10.3390/vaccines7030089

**Published:** 2019-08-14

**Authors:** Mansi Sharma, Florian Krammer, Adolfo García-Sastre, Shashank Tripathi

**Affiliations:** 1 Department of Microbiology & Cell Biology, Indian Institute of Science, Bengaluru 560012, India; 2 Centre for Infectious Disease Research, Indian Institute of Science, Bengaluru 560012, India; 3 Department of Microbiology, Icahn School of Medicine at Mount Sinai, New York, NY 10029, USA; 4 Global Health and Emerging Pathogens Institute, Icahn School of Medicine at Mount Sinai, New York, NY 10029, USA; 5 Department of Medicine, Division of Infectious Diseases, Icahn School of Medicine at Mount Sinai, New York, NY 10029, USA; 6 The Tisch Cancer Institute, Icahn School of Medicine at Mount Sinai, New York, NY 10029, USA

**Keywords:** omics, systems biology, systems vaccinology, vaccine, signature, biomarkers, immunogenicity, protection, adjuvant, antigen, microbiome

## Abstract

An ideal vaccine provides long lasting protection against a pathogen by eliciting a well-rounded immune response which engages both innate and adaptive immunity. However, we have a limited understanding of how components of innate immunity, antibody and cell-mediated adaptive immunity interact and function together at a systems level. With advances in high-throughput ‘Omics’ methodologies it has become possible to capture global changes in the host, at a cellular and molecular level, that are induced by vaccination and infection. Analysis of these datasets has shown the promise of discovering mechanisms behind vaccine mediated protection, immunological memory, adverse effects as well as development of more efficient antigens and adjuvants. In this review, we will discuss how systems vaccinology takes advantage of new technology platforms and big data analysis, to enable the rational development of better vaccines.

## 1. Introduction

Vaccines are one of the greatest breakthroughs in the field of Medicine and have saved and improved human lives on a tremendous scale across the globe. Yet most of the current successful vaccines were developed empirically through an ‘isolate, inactivate or attenuate, and inject’ approach [1,2]. Even with technological advances and extensive knowledge of the human immune system, we still have a limited understanding of what ensures that a vaccine will be successful. Development of effective vaccines against important viral pathogens such as human immunodeficiency virus (HIV), influenza and dengue viruses remain challenging. The major hurdles include identifying early markers of vaccine efficacy or adverse reactions, developing relevant antigens and adjuvants, defining correlates of protection and understanding mechanisms underlying long-lasting protective immune responses generated by vaccination. The immune response to vaccines is highly complex, multifactorial and greater than the sum of the parts. It involves multi-level interaction networks, linking intra-cellular biochemical signaling pathways, inter-cellular communication and inter-organ cellular trafficking through space and time. These interactive networks have emergent properties such as immunological memory and protection from disease, which cannot be delineated by conventional reductionist approaches [3].

The rapid emergence of high-throughput technology platforms in biology and the use of systems-based approaches to analyze and integrate large and varied sets of Omics data holds the promise of providing broader and deeper understanding of these complex phenomena [4]. Systems biology involves (i) monitoring different components of the biological system in response to specific perturbations, (ii) integration of multiple types of data over time, and (iii) creation of mathematical models to predict the structure and behavior of the system in question.

**Figure 1:**
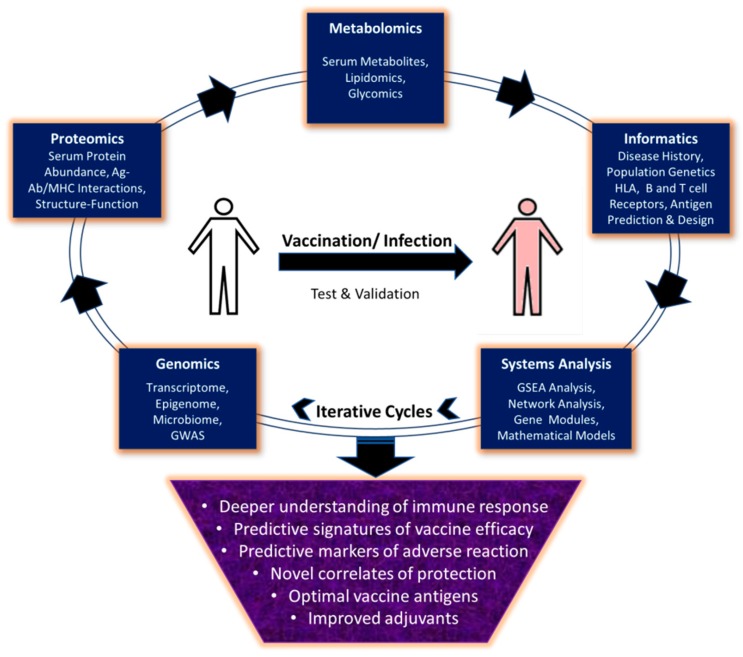
Applications of Omics methodologies and systems biology in vaccine development.

Systems vaccinology is an emerging subdiscipline of systems biology, which aims to reconstruct a comprehensive view at the organism level, of the dynamic responses to vaccines through measurement of a multitude of Omics data types sampled in parallel. It requires the testing and validation of novel hypotheses and insights that may arise from the first sets of data analysis and subsequent iterative cycles of experimentation to improve predictive models (Figure 1). In the following sections we will discuss how the use of high-throughput methodologies and systems vaccinology should enable the transition from empirical towards rational vaccine design, especially in the context of viral pathogens.

## 2. Antigen Discovery and Development

Traditionally, mapping of immunodominant B cell epitopes required identification of the whole protein first, followed by the assessment of its fragments for reactivity with antibodies. This approach to antigen discovery and epitope mapping is time consuming, labor intensive and expensive. Advances in Omics approaches, such as protein microarrays to map the reactivity of antibodies and T cells to antigens, availability of various databases related to host–pathogen protein–protein interactions, tools for structural analyses and computational capabilities for theoretical predictions have tremendously helped in antigen discovery [5,6,7]. In particular, the in silico analytical and predictive methods have greatly facilitated all aspects of biological research including vaccinology. The approach of reverse vaccinology has become an integral part of rational vaccine design. This involves computational mining and analysis of large datasets related to immune responses to pathogens, host-pathogen interactions and host and pathogens genomes to predict promising antigen candidates. This entails large-scale sampling of potential antigens and down selection based on affinity for the antibodies or major histocompatibility complex (MHC) molecules for experimental validation.

Structural vaccinology is another approach which is facilitating rational design of better antigens. The availability of structures of viral proteins as well as antigen-antibody complexes has made it possible to carry out docking and modeling studies for prediction of B cell epitopes. This approach has been used to develop strongly immunogenic vaccine candidates from respiratory syncytial virus (RSV) glycoprotein and middle east respiratory syndrome (MERS) virus spike protein [8,9]. High-throughput in vitro assays combined with structure-function analyses have been successfully used to discover broadly neutralizing antibodies (bNAbs) against influenza HA stalk, HIV V2 and V3 glycans and Dengue envelope protein [10,11,12]. Structural information of bNAbs-antigen complexes has been used to graft linear as well as discontinuous HIV epitopes on to computationally designed scaffolds to develop designer antigens [13,14]. NGS-based profiling of antibody repertoire induced by such antigens can help in identification of bNAbs and improved antigens. By using this methodology, improved vaccine candidates against RSV have been developed and bNAbs against HIV glycoprotein have been discovered [14,15,16]. Combining orthogonal datasets related to immune response to vaccines can also yield information about key antibody targets. Using this approach, Lee et al. performed a high-resolution proteomics analysis of immunoglobulin coupled with high-throughput sequencing of transcripts encoding B cell receptors. This allowed quantitative estimation of antibody repertoire at the individual level before and after vaccination with trivalent seasonal influenza vaccine [17], and identification of HA-head specific broadly protective antibodies.

In comparison to B cell antigens (mostly conformational), T cell epitopes (mostly linear) are relatively simpler to predict, as the parameters required for data mining are based on the properties of interaction between MHC proteins and the antigenic peptides. Although extensive diversity in MHC haplotypes at individual level presents a major challenge in identifying broadly protective T cell epitopes, it can be compensated to a significant degree by including most frequently present HLA alleles across various populations in the epitope prediction protocol. The simplicity of peptides makes them an attractive target for vaccine design; however, T cell-epitope based design has not been able to deliver any commercial vaccine yet. This is because a standalone exogenous recombinant T cell antigen-based vaccine will not elicit CD8 positive T cell response, unless cross-presentation is engaged. This limitation has been overcome significantly by vaccines based on viral vectors, DNA vaccines and use of adjuvants that facilitate cross-presenting dendritic cell recruitment. Furthermore, correlates of cell mediated protection are still not well defined, and a combination of B and T cell-mediated immunity might always be needed to counter most viral pathogens.

## 3. Molecular Signatures of Vaccine Efficacy

Vaccine-mediated protection is generated through a combination of antibody and cellular immunity; however, current methods of predicting vaccine efficacy primarily rely on measuring the antibody quantity and quality for several reasons: (i) Antibodies, and not cellular immunity, have the potential to mediate sterile immunity (protection from infection); (ii) the vaccines are primarily targeted towards eliciting a B cell response; and (iii) although T cell responses are elicited by many vaccines, reliable standardized assays to predict the protective efficacy of cellular immunity are still not available. With the use of Omics methodologies to measure systems level changes induced by vaccines, molecular signatures of efficacy can be predicted. Such studies have now been conducted for the yellow fever vaccine [18]. This vaccine consists of a live-attenuated strain (YF-17D) of the yellow fever virus that induces both neutralizing antibodies as well as potent and long-lived CD8+ T cell responses. Using microarray gene expression profiling, induction of an interferon and innate antiviral gene signature was detected post vaccination in peripheral blood mononuclear cells (PBMCs) [19]. By adding multiparameter flow cytometry data, activation of innate immune pathways was analyzed through computational modeling and unique gene signatures for predicting the induction of efficient CD8+ T cell and neutralizing antibody responses were identified. The predictive CD8+ T cell signature included the expression of complement protein C1qB and the eukaryotic translation initiation factor 2 alpha kinase 4 (EIF2AK4), which is an orchestrator of the integrated stress response. Meanwhile the B cell growth factor receptor TNFRSF17 was among the genes that emerged in the antibody response signature [20].

Following this initial study, systems biology approaches have been applied to study immune responses to vaccines against a wide range of pathogens, including influenza virus, smallpox, and HIV [21,22,23]. The study of transcriptional signatures induced by inactivated trivalent influenza vaccine (TIV) on day 7 post vaccination revealed markers associated with expansion of plasmablasts and the unfolded protein response in B cells, which were predictive of influenza virus-specific antibody responses on day 28 [21]. Interestingly, TNFRSF17, which was predictive of antibody responses to YF-17D, also appeared in the signatures predictive of TIV response. A critical question is whether there are universal signatures capable of predicting antibody responses to different kinds of vaccines. To this end, systems-based approaches were used to compare signatures induced by different types of vaccines YF-17D, live attenuated influenza vaccine (LAIV), TIV, the carbohydrate meningococcal vaccine (MPSV4), and the conjugate meningococcal vaccine (MCV4) [24]. Specifically, gene expression data in human blood were curated from over 500 studies and 30,000 expression profiles and a master network was created. From this, 334 Blood Transcriptional Modules (BTMs) were curated. The study concluded that antibody response to inactivated vaccines (e.g., seasonal influenza virus vaccine, diphtheria toxoid component of the conjugate meningococcal vaccine) are associated with transcriptional modules related to plasmablast differentiation, whereas the antibody responses of live-attenuated vaccines (e.g., yellow fever vaccine) are highly correlated with modules involving innate immunity and type I interferon responses. Thus, signatures of immunity may vary with the class of vaccine [24]. The ‘immunologic signatures’ representing a broad range of cell states and perturbations within the immune system have been compiled in MSigDB, which is a collection of annotated gene sets for use with gene set enrichment tools [25].

Omics approaches allow monitoring of systemic changes at molecular, cellular and organism level in response to vaccination or infection. These approaches include genomics, proteomics and metabolomics, which allow study of parameters shown in the figure in respective boxes. Integration of these diverse systems level datasets using computational tools and mathematical models allows prediction of markers of protection or adverse reactions to vaccines, development of improved vaccine antigens and adjuvants. However, these predictions require validation through iterative cycles of experimentation to get conclusive evidence and inclusion in vaccine formulation.

Integration of different types of Omics data can give further insight into molecular mechanisms underlying vaccine efficacy. Franco et al. searched for genetic and transcriptional components associated with the magnitude of antibody immune response to influenza vaccination, through genome-wide single nucleotide polymorphism genotyping and transcriptional profiling. They were able to map expression quantitative trait loci (eQTL) that could be important determinants of vaccine immunogenicity [26]. Integration of metabolomics into models of vaccine immunity can reveal the link between transcriptional events and biological mechanisms. To this end, Shuzhao et al. studied the response to varicella zoster vaccine (Zostavax) by measuring transcriptomic and metabolomic changes upon vaccination [27]. Further integration of orthogonal datasets related to transcription and metabolite changes in cell populations and cytokine levels allowed them to create a multiscale, multifactorial response network (MMRN) of immunity. They found that networks associated with inositol phosphate, glycerophospholipids and sterol metabolism, especially the sterol regulatory binding protein 1, are key predictors of antibody and T cell responses. This approach is broadly applicable to study vaccine responses and identify predictors of efficacy.

## 4. Predictive Markers of Adverse Effects

Adverse reactions to vaccine candidates pose a major hurdle in regulatory approval. Although mild reaction such as transient fever and local swelling at the injection site are fairly common, severe adverse reactions have also been reported in a few rare instances. Fatal viscerotropic disease caused by yellow fever vaccination (1 in 250,000 cases) and cases of narcolepsy in 2009 H1N1 pandemic vaccinees are a few examples. Immunological characterization of patients with viscerotropic disease showed 200-fold elevated levels of CD14+CD16+ inflammatory monocytes [28]. Genetic background studies of narcoleptic patients showed association of symptoms with ethnic background including the HLA-DQB1*06:02 genotype [29]. These examples show that it is possible to identify clinical and genetic markers that may lead to adverse reaction to a specific vaccine. Since such cases are very rare, it is very important to have the appropriate clinical infrastructure and surveillance to identify such cases and follow them over time to collect samples for analysis in order to identify molecular mechanisms and predictive markers of severe adverse reactions.

## 5. Rational Use and Development of Adjuvants

Viral pathogens express pathogen associated molecular patterns (PAMPS) that are detected by cellular pattern recognition receptors (PRRs) in mammalian cells. They trigger cellular innate immune responses that build up an antiviral state and subsequently lead to induction of genes that are key mediators of immune cell recruitment and the development of adaptive immunity [3]. For subunit or inactivated vaccines that lack viral PAMPS, adjuvants are critical to fulfill the requirements of eliciting the appropriate innate immune responses. This opens the possibility of designing ligands that stimulate specific PRRs in order to get the desired type and intensity of adaptive immune response against specific pathogens. In silico designing, homology modeling, molecular docking and *in vitro* screening approaches have been used to develop ligands for Toll like receptors (TLRs) to enhance antigen presenting cell recruitment [30]. Similarly, CCR4 receptor antagonists have been developed to enhance T cell and antibody responses and CD1d agonists to activate natural killer T cells [31,32].

The current understanding of adjuvant function is that they induce controlled inflammation and recruitment of antigen presenting cells. However, details of how this leads to specific types of adaptive immunity are not completely understood. For example, for the most commonly used adjuvant, alum, the molecular events leading to enhanced immune response are still not clear, although the NALP3 inflammasome has been implicated [33]. After alum, MF59 was the first adjuvant used in licensed human vaccines and shown to elicit strong antibody responses to co-administered antigens [34]. MF59 has been reported to enhance the diversity and affinity of the antibody response as well as the longevity of protection elicited following influenza vaccination in humans [35]. This effect is also attributed to enhanced recruitment and activation of antigen-presenting cells that stimulate vaccine-specific CD4+T cells, leading to the induction of specific antibodies targeting broader neutralizing epitopes [36]. Comparison of the transcriptional profile induced in mice in response to alum and MF59 shows three-times as many genes induced by MF59 than by alum at the injection site, including the transcription factor Jun B, and suggestive of an effect through type I IFN independent mechanisms [37,38]. Similarly, the GSK-adjuvants AS03 (contains α-tocopherol) and AS04 (contains TLR4 ligand) have also been shown to induce stronger transcriptional responses through the NFkB pathway leading to higher expression of immune cell-recruiting chemokines and pro-inflammatory cytokines than alum [39,40,41]. Transcriptional profiling of human subjects in response to HIV-1 envelope vaccine adjuvanted with TLR4 agonist glucopyranosyl lipid revealed BTMs, related to innate immune cell activation at early time points and T and B cell activation at the later time points post-immunization [42]. Recently, unique and shared transcriptional profiles of alum, CAF01, IC31 and GLA-SE, were studied using genome-wide transcriptomic analysis of whole blood (WB) and draining lymph nodes (dLNs) in mice at early time points post immunization [43]. Large variations in transcriptional profile, both in magnitude and kinetics, were observed among these adjuvants, with alum and GLA-SE inducing the least and greatest transcriptional responses, respectively.

As discussed here, systems-based approaches to understanding the basis of adjuvanticity have been limited mostly to transcriptional data sets. This approach should be extended to proteomic and metabolomics data sets obtained from individuals vaccinated with or without adjuvants. Integration of this information can inform the optimal use of the existing vaccine adjuvants, as well as the development of rationally engineered adjuvants for human vaccines.

## 6. Conclusions and Prospective

With advances in Omics methodologies, it is now possible to obtain global readouts of cellular and molecular events, leading to a more comprehensive understanding of the immune responses to vaccines and pathogens. Systems-level understanding of vaccine-induced immunity will require integration of increasingly accessible varied data types. This will require continued efforts towards development of open access computational tools for epitope prediction, databases related to host–pathogen interactions and omics data analyses. Several selected online resources related to this subject have been listed in Table 1. Furthermore, it is crucial to standardize technology platforms, data acquisition and analysis methodologies to improve reproducibility of the Omics data and allow inter-lab comparisons. Management of standardized Omics datasets for easy access to scientific community, which enables better integration and meta-analysis, is critical. Furthermore, systems analysis is mostly useful for generating hypotheses that require validation in relevant *in vitro* and *in vivo* models and, subsequently, in clinical studies. If successful, discovery and use of the molecular signatures of protection or adverse effects of new vaccines can save resources, accelerate vaccine research and development, reduce the length and cost of clinical trials, and lead to enhanced pandemic and epidemic preparedness. So far, systems-level analysis of immune responses to vaccines has generally depended on datasets generated from PBMCs, molecular measurements from serum and profiling of immune cell populations. Moving forward, additional parameters such as age, sex, ethnicity, pre-existing immunity, diseases such as diabetes, obesity and chronic infections, stress, nutrition, microbiome, environmental and socioeconomic factors should be considered, as well. With all these parameters, systems vaccinology and use of Omics methodologies holds the promise of personalized vaccine development, i.e., giving the right vaccine to the right person, at the right dose, through the right route and at the right time. To deliver all the promises and realize the full potential of systems vaccinology, immunologists, virologists, computational biologists and mathematicians must work together to move towards next generation of rationally designed viral vaccines.

## Figures and Tables

**Table 1:** Online resources for application in vaccine design and development.

Online Resource	Application & URL	References
**Antigen Prediction**		
IEDB	http://www.iedb.org/	Zhang et al., 2008 [44,45]
EpiJen	http://www.ddg-pharmfac.net/epijen/EpiJen/EpiJen.htm	Doytchinova et al., 2006 [46]
MULTIPRED2	http://cvc.dfci.harvard.edu/multipred2/index.php	Zhang et al., 2011 [47]
Propred	http://crdd.osdd.net/raghava/propred/	Singh et al., 2001 [48]
Bcepred	http://crdd.osdd.net/raghava/bcepred/	Saha et al., 2004 [49]
**Gene Set Enrichment and Network Analysis**		
Metascape	http://metascape.org/gp/index.html#/main/step1	Tripathi et al., 2015 [50]
Reactome	https://reactome.org/	Joshi-Tope et al., 2005 [51]
Cytoscape	https://cytoscape.org/	Shannon et al., 2003 [52]
DAVID	https://david.ncifcrf.gov/	Jiao et al., 2011 [53]
**Data repositories and Analysis tools**		
ImmPort	https://www.immport.org/home	Bhattacharya et al., 2014 [54]
Immunespace	https://www.immunespace.org/	Brusic et al., 2014 [55]
10,000 Immunomes	http://10kimmunomes.ucsf.edu/	Zalocusky et al., 2018 [56]
MSigDB	http://software.broadinstitute.org/gsea/msigdb	Liberzon et al., 2011 [25]
Gene Expression Omnibus	https://www.ncbi.nlm.nih.gov/geo/	Edgar et al., 2002 [57]
